# YKL-40 Level Is Associated with TyG-BMI-Estimated Insulin Resistance and Metabolic Syndrome in a Population Without Diabetes, Independent of Obesity

**DOI:** 10.3390/ijms26199682

**Published:** 2025-10-04

**Authors:** Hsin-Hua Chou, Shing-Hsien Chou, Kuan-Hung Yeh, Hsuan-Li Huang, I-Shiang Tzeng, Yu-Lin Ko

**Affiliations:** 1 Division of Cardiology, Department of Internal Medicine, Taipei Tzu Chi Hospital, Buddhist Tzu Chi Medical Foundation, New Taipei City 23142, Taiwan; hhchou@tzuchi.com.tw (H.-H.C.); yeh2644@tzuchi.com.tw (K.-H.Y.); tch04494@tzuchi.com.tw (H.-L.H.); 2 School of Medicine, Tzu Chi University, Hualien 97037, Taiwan; 3 Division of Cardiology, Chang Gung Memorial Hospital, Taoyuan 33305, Taiwan; n12323@cgmh.org.tw; 4 Graduate Institute of Clinical Medical Sciences, College of Medicine, Change Gung University, Taoyuan 33332, Taiwan; 5 Department of Research, Taipei Tzu Chi Hospital, Buddhist Tzu Chi Medical Foundation, New Taipei City 23142, Taiwan; xdd05082@tzuchi.com.tw

**Keywords:** YKL-40, TyG-BMI, insulin resistance, metabolic syndrome, obesity

## Abstract

YKL-40, an obesity-related inflammatory biomarker, has inconsistently been associated with insulin resistance, and its relationship with metabolic syndrome is not well established. This study investigated the associations of YKL-40 levels with insulin resistance and metabolic syndrome independently of obesity. We analyzed data from 4303 participants without diabetes in the Taiwan Biobank. Insulin resistance was defined by the highest quartile of triglyceride-glucose body mass index (TyG-BMI). Metabolic syndrome was defined per AHA/NLHBI criteria. Both univariate and multivariate analyses demonstrated significant correlations between YKL-40 levels and TyG-BMI. Participants with higher YKL-40 quartiles exhibited increased odds of TyG-BMI-estimated insulin resistance even after adjusting for established predictors of TyG-BMI, including waist circumference. Similarly, higher YKL-40 quartiles significantly correlated with increased metabolic syndrome prevalence, and this relationship persisted after stratifying participants by weight status (normal weight vs. overweight/obese). Interaction analysis indicated that overweight/obesity individuals consistently had higher metabolic syndrome prevalence than normal-weight counterparts within identical YKL-40 quartiles, though the impact of overweight/obese diminished across rising YKL-40 quartiles (*p* for interaction = 0.008). Increased YKL-40 levels are significantly associated with TyG-BMI-estimated insulin resistance and metabolic syndrome, independent of obesity. There is a significant interaction between overweight/obese and YKL-40 levels in determining metabolic syndrome prevalence.

## 1. Introduction

Obesity has become a global epidemic due to the increased availability of calorie-dense foods and reduced physical activity, imposing a heavy burden on public health and quality of life. Over the past three decades, global overweight and obesity rates have nearly doubled [1], making obesity a major contributor to disability and mortality.

Obesity is a multifactorial disorder caused by chronic positive energy balance. Surplus calories are stored as triglycerides in adipose tissue, which expands through hypertrophy and hyperplasia. When subcutaneous fat storage is exceeded, ectopic fat accumulates in visceral compartments, including the peritoneum, liver, myocardium, skeletal muscle, and pancreas [2]. These enlarged adipocytes and ectopic depots release free fatty acids, reactive oxygen species, and proinflammatory cytokines, promoting insulin resistance and type 2 diabetes mellitus (T2DM) [3,4].

Metabolic syndrome is characterized by a cluster of interrelated cardiometabolic risk factors, including central obesity, elevated blood pressure, hyperglycemia, reduced high-density lipoprotein cholesterol (HDL-C) levels and elevated triglyceride levels. It is recognized as a global health issue due to its association with increased risks of cardiovascular disease and T2DM [5,6]. Visceral obesity and insulin resistance are the main drivers of metabolic syndrome, with physical inactivity, aging, and hormonal imbalances as additional contributors [5]. Moreover, growing evidence suggests that chronic low-grade inflammation plays a central role in insulin resistance and metabolic syndrome [7,8].

YKL-40 (chitinase-3-like-1) is an inflammatory biomarker secreted by various human cell types such as neutrophils, activated macrophages, vascular smooth muscle cells and cancer cells [9,10,11,12,13]. Adipose tissue is a major source of circulating YKL-40 [14,15,16]. While studies have linked YKL-40 with insulin resistance [13,17,18], the findings are inconsistent [15,19]. Its relationship with metabolic syndrome has been poorly investigated; only one small cross-sectional study reported a significant association [20]. It is also unclear whether YKL-40 independently associates with insulin resistance and metabolic syndrome or simply reflects obesity.

Therefore, this study aimed to examine the associations of YKL-40 with triglyceride-glucose body mass index (TyG-BMI)-estimated insulin resistance and metabolic syndrome and to determine whether these associations are independent of obesity in a non-diabetic population.

## 2. Results

### 2.1. Baseline Characteristics of Participants by YKL-40 Quartiles

Table 1 summarizes the baseline characteristics of the participants according to YKL-40 quartiles. Participants in the higher YKL-40 quartiles were generally older, more likely to report regular exercise habits, and exhibited greater waist circumference (WC), systolic blood pressure (SBP), and diastolic blood pressure (DBP). They also exhibited significantly elevated fasting plasma glucose, glycated hemoglobin (HbA1C), total cholesterol, and triglyceride, along with notably lower HDL-C. Interestingly, no significant differences were found in body mass index (BMI) across the YKL-40 quartiles. Moreover, participants in the higher YKL-40 quartiles had elevated TyG-BMI and a higher prevalence of metabolic syndrome, along with its individual components.

### 2.2. Predictors of TyG-BMI: Univariate and Multivariate Analysis

Table 2 presents the results of stepwise linear regression analyses designed to identify predictors of TyG-BMI. In univariate analyses, age, gender, smoking status, WC, SBP, DBP, HDL-C, low-density lipoprotein (LDL)-C and YKL-40 levels showed significant associations with TyG-BMI. These variables were subsequently incorporated into the multivariate regression model. Except for smoking status, all variables retained significance as independent predictors. Among these, WC was identified as the strongest predictor, followed by HDL-C, DBP, male gender, LDL-C, YKL-40 levels, age and SBP.

### 2.3. Relationship Between YKL-40 Levels and TyG-BMI-Estimated Insulin Resistance

Table 3 demonstrates the relationship between YKL-40 quartiles and TyG-BMI-estimated insulin resistance. Participants in the higher YKL-40 quartiles exhibited a significant greater prevalence of TyG-BMI-estimated insulin resistance. This relationship remained significant even after controlling baseline characteristics and known predictors of TyG-BMI, including WC.

### 2.4. Association Between Ykl-40 Levels and Metabolic Syndrome

**Figure 1:**
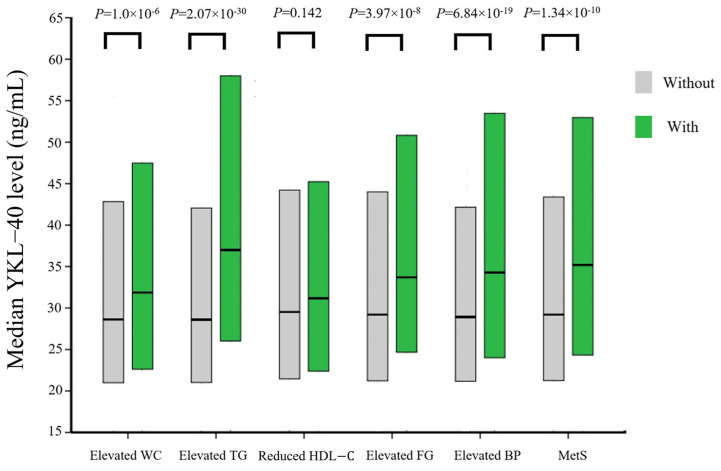
Comparison of the median YKL-40 levels in participants with and without metabolic syndrome and its individual component. Box plots showed the median, 25th, and 75th percentiles of YKL-40 levels. Abbreviations: WC, waist circumference; TG, triglycerides; HDL-C, high-density lipoprotein cholesterol; FG, fasting glucose; BP, blood pressure; MetS, metabolic syndrome.

Figure 1 compares YKL-40 levels between participants with and without metabolic syndrome and across individual metabolic syndrome components. Median YKL-40 levels were significantly higher in participants diagnosed with metabolic syndrome and those exhibiting each component, except for reduced HDL-C levels.

Table 4 presents the association between YKL-40 quartiles and the prevalence of metabolic syndrome. Participants in higher YKL-40 quartiles demonstrated a significantly increased prevalence of metabolic syndrome. This association persisted after adjustment for age, sex, smoking status and exercise habits. Further stratification into normal weight (BMI < 23 kg/m^2^) and overweight/obesity (BMI ≥ 23 kg/m^2^) groups consistently showed an increased prevalence of metabolic syndrome among those in the higher YKL-40 quartiles in both groups.

### 2.5. Influence of Obesity on the Association Betweenykl-40 Quartiles and Metabolic Syndrome

**Figure 2:**
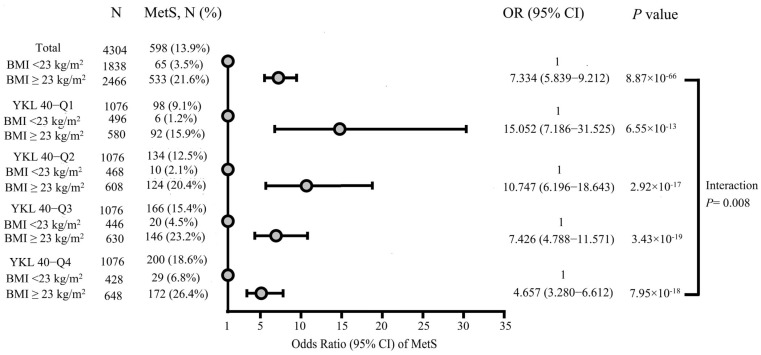
Interaction between overweight/obesity and YKL-40 quartiles in relation to the prevalence of metabolic syndrome, adjusted for age, sex, smoking status and exercise habits. Dots indicate the odds ratios of metabolic syndrome. Abbreviations: BMI, body mass index; MetS, metabolic syndrome; OR, odds ratio; CI, confidence interval.

Figure 2 assesses the modifying effect of overweight/obesity on the association between YKL-40 quartile and metabolic syndrome. Overweight/obesity participants exhibited a significant higher prevalence of metabolic syndrome compared to their normal-weight counterparts across all YKL-40 quartiles. Notably, the effects of overweight/obese on the prevalence of metabolic syndrome diminished progressively from the lowest to the highest YKL-40 quartiles (lowest quartile: odds ratio OR [odd ratio], 15.052; 95% CI [confidence interval], 7.186–31.525; highest quartile: OR, 4.657; 95% CI, 3.280–6.612). The interaction between overweight/obesity and YKL-40 quartiles on metabolic syndrome prevalence was statistically significant (*p* for interaction = 0.008)

## 3. Discussion

In the current study, elevated YKL-40 levels exhibited significant associations with multiple cardiometabolic risk factors, including increased WC, elevated blood pressure, higher fasting blood glucose, increased glycated hemoglobin, and dyslipidemia. Higher YKL-40 levels also correlated significantly with increased TyG-BMI, metabolic syndrome, and its individual components. Participants in the higher quartiles of YKL-40 demonstrated a greater prevalence of TyG-BMI-estimated insulin resistance independent of WC and established predictors of TyG-BMI. Additionally, these individuals also exhibited an increased prevalence of metabolic syndrome, and the association between YKL-40 quartiles and metabolic syndrome prevalence remained significant after stratifying participants into normal weight and overweight/obese groups. Notably, we also identified a significant interaction between overweight/obesity and YKL-40 quartiles in relation to the prevalence of metabolic syndrome, indicating that the impact of overweight/obesity on the risk of developing metabolic syndrome was markedly attenuated among individuals in the higher YKL-40 quartiles.

### 3.1. YKL-40 Levels and Insulin Resistance

The development of insulin resistance is influenced by both genetic predisposition and environmental factors, with central obesity recognized as the most common etiological contributor [21]. In obesity-related insulin resistance, dysfunctional adipose tissue releases free fatty acids, reactive oxygen species, and elevated proinflammatory cytokines, all of which impair insulin sensitivity and disturb glucose homeostasis. Among individuals with obesity and T2DM, circulating YKL-40 levels correlate with increased YKL-40 mRNA and protein expression in visceral adipose tissue, highlighting the pivotal role of visceral fat in regulating YKL-40 expression [14]. Furthermore, several studies have demonstrated a reduction in YKL-40 levels following weight loss in individuals with obesity [14,17]. Consistent with these findings, accumulating evidence supports a strong association between YKL-40 levels and insulin resistance [13,17,18]. Nevertheless, some studies have reported conflicting results and failed to identify significant associations [15,19]. Additionally, Nielsen et al. noted that plasma YKL-40 levels were unrelated to obesity-related parameters and identified YKL-40 as an obesity-independent marker of T2DM, being linked instead to fasting plasma glucose and plasma IL-6 levels [16]. These inconsistencies may be attributed to small sample sizes and the inclusion of patients with T2DM in earlier studies.

In the current investigation, we examined the relationship between YKL-40 levels and TyG-BMI-estimated insulin resistance in a large cohort of individuals without diabetes. Participants in the higher YKL-40 quartiles exhibited significantly greater WC, but not BMI, underscoring the contribution of visceral adiposity to circulating YKL-40 levels. In linear regression analyses, YKL-40 levels were strongly associated with TyG-BMI, and this association remained robust after adjustment for potential confounders, including WC. Furthermore, the prevalence of TyG-BMI-estimated insulin resistance was significantly greater among participants in the higher YKL-40 quartiles. This association persisted even after controlling baseline characteristics, blood pressure, lipid parameters, and WC, suggesting that YKL-40 may serve as a biomarker for TyG-BMI-estimated insulin resistance independent of obesity.

Although adipose tissue is widely considered a major source of YKL-40 secretion, other factors such as genetic predisposition or diseases characterized by inflammation may also contribute. It is plausible that, as an inflammatory biomarker, elevated YKL-40 might promote the development of insulin resistance even in individuals with normal weight. Nevertheless, further studies are needed to elucidate the mechanisms through which YKL-40 directly influences insulin resistance.

### 3.2. YKL-40 Levels with Cardiometabolic Risk Factors and Metabolic Syndrome

Previous studies have shown that elevated YKL-40 levels are associated with dyslipidemia and blood pressure. In a prospective Danish cohort of 21,647 participants, individuals in the top YKL-40 decile (91st to 100th percentile) had a 34% increase in triglyceride levels compared to those in the lowest tertile (0th to 33rd percentile). However, no significant association was found with cholesterol levels, although modest correlations were observed with HDL-C and LDL-C [22]. In the Danish MONICA study, which included 2656 participants, both cholesterol and triglyceride levels increased significantly across higher YKL-40 quartiles. LDL-C levels increased slightly from the first to the third quartile, while HDL-C remained relatively unchanged [23].

As an inflammatory biomarker, YKL-40 modulates differentiation, proliferation, and migration of vascular smooth muscle cells, as well as chemotaxis and migration of vascular endothelia cell [10,13,24]. These biological processes provide plausible mechanisms link elevated YKL-40 levels to hypertension and adverse cardiovascular outcomes. In the CLARICOR trial, which enrolled 4298 patients with stable coronary artery disease, hypertensive individuals exhibited significantly higher YKL-40 levels [25]. Over a 2.6-year follow-up, elevated serum YKL-40 predicted increased risks of myocardial infarction, cardiovascular death, and all-cause mortality. In a Chinese case–control study involving 700 matched pairs of new-onset hypertension cases and controls, men in the higher YKL-40 tertiles exhibited an increased risk of hypertension [26]. Additionally, Bakirci et al. further observed that YKL-40 levels were markedly higher in patients with non-dipping hypertension patterns [27].

Given its robust associations with central obesity, insulin resistance, dyslipidemia, and elevated blood pressure, one would anticipate YKL-40 to correlate with metabolic syndrome prevalence. Yet, only a single small-scale study has found a significant relationship between YKL-40 levels and metabolic syndrome to date [20]. Furthermore, as an inflammatory biomarker closely tied to obesity, it remains unclear whether YKL-40 is independently associated with metabolic syndrome or merely acts as an epiphenomenon of obesity and metabolic syndrome. In our current study, individuals in the highest YKL-40 quartiles exhibited significantly higher systolic and diastolic blood pressure, elevated triglyceride and total cholesterol levels, findings that are consistent with prior reports. Prevalence of both individual metabolic syndrome components and the syndrome overall increased across YKL-40 quartiles, except for reduced HDL-C. In line with these findings, the median YKL-40 level was significantly greater in those diagnosed with metabolic syndrome and its individual components. Cox regression analysis further confirmed that higher YKL-40 quartiles were significantly associated with increased prevalence of metabolic syndrome. Importantly, this association persisted after stratifying participants into normal weight (BMI < 23 kg/m^2^) and overweight/obesity (BMI ≥ 23 kg/m^2^) groups, suggesting that the association between YKL-40 and metabolic syndrome is independent of obesity. Notably, as YKL-40 quartiles increased, the impact of overweight/obese on the risk of metabolic syndrome attenuated, indicating a significant interaction effect between overweight/obesity and YKL-40 quartiles in relation to metabolic syndrome prevalence. This suggests that in participants with elevated YKL-40 levels, a heightened inflammatory status may independently drive insulin resistance, hyperglycemia, dyslipidemia, and hypertension, thereby attenuating adiposity’s relative contribution to the development of metabolic syndrome.

### 3.3. Clinical Implications

The present study demonstrates a significant association between YKL-40 levels with TyG-BMI-estimated insulin resistance and metabolic syndrome, independent of obesity. YKL-40 can be easily measured using commercially available, low-cost enzyme-linked immunosorbent assay (ELISA) kits. Incorporating YKL-40 levels into risk stratification may be cost-effective, particularly for normal-weight individuals who might otherwise be overlooked in preventive cardiometabolic care. Moreover, accumulating evidence suggested that elevated YKL-40 levels can prognosticate unfavorable long-term outcomes in patients with coronary artery disease [28,29]. Notably, this prognostic value appears to be independent of C-reactive protein levels [29]. The strong associations observed in our study provide further insight into the potential role of YKL-40 as a predictive biomarker for adverse cardiovascular outcomes.

### 3.4. Study Limitations

While our study revealed a strong association between YKL-40 levels and both insulin resistance and metabolic syndrome independent of obesity in a large cohort, its non-prospective study design prevents establishing causality. Due to the lack of insulin measurements and homeostatic Model Assessment of Insulin Resistance (HOMA-IR) data in the original TWB database, we used TyG-BMI as a surrogate marker for insulin resistance. Nonetheless, growing evidence supports TyG-BMI as a reliable surrogate marker of insulin resistance, with individuals in its highest quartile exhibiting an increased risk of developing T2DM. Moreover, information on additional inflammatory biomarkers, including C-reactive protein, was unavailable in the TWB dataset. It remains unclear whether the observed relationships between YKL-40 levels and insulin resistance or metabolic syndrome are independent of other inflammatory biomarkers. Finally, because our cohort exclusively comprised individuals of Han Chinese descent, these findings may not be generalized to other ethnic groups. Future studies involving multi-ethnic populations, prospective study designs, direct measurement of insulin levels to define insulin resistance, and adjustment for additional inflammatory biomarkers are warranted to validate these associations and further elucidate the pathophysiological mechanisms linking YKL-40 to insulin resistance and metabolic syndrome.

## 4. Materials and Methods

### 4.1. Study Participants and Design

The study cohort was drawn from the Taiwan Biobank (TWB), which recruited Taiwanese adults aged 30–70 years, without any history of cancer, from multiple centers across Taiwan between 2008 and 2015. The TWB is a national resource containing data on blood and urine analyses, genetic profiles, baseline demographic information, and lifestyle-related questionnaire responses. All participants provided written informed consents and self-reported Han Chinese ethnicity.

**Figure 3:**
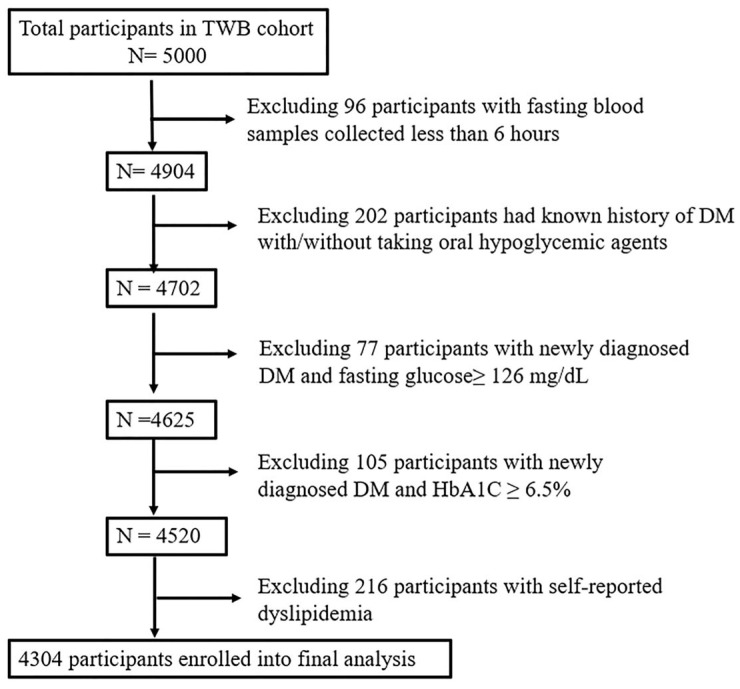
Flowchart of the study participants. Abbreviations: DM, diabetes mellitus; HbA1C, glycated hemoglobin; TWB, Taiwan Biobank.

Initially, 5000 participants were enrolled in this study. Ninety-six participants were subsequently excluded due to blood samples being collected within 6 h of fasting. Additionally, 384 participants were excluded due to either a known history (n = 202) or a newly diagnosed (n = 182) T2DM. To minimize the confounding effects of lipid-lowering medications on lipid profiles, another 216 participants who self-reported dyslipidemia were also excluded. Ultimately, 4304 participants remained for the final analysis (Figure 3). Ethical approval for the study was granted by the Research Ethics Committee of Taipei Tzu Chi Hospital, Buddhist Tzu Chi Medical Foundation (approval number: 05-X04-007), and the Ethics and Governance Council of Taiwan Biobank (approval number: TWBR11108-01).

### 4.2. Demographic Data and Laboratory Examinations

Demographic and biochemical data were sourced from the TWB database [30]. Recorded demographic variables included age, sex, height, weight, BMI, WC, SBP, and DBP. Participants were classified as having regular exercise habits if they answered “yes” to either of the following: (1) exercising for at least 30 min on three or more days per week or (2) engaging in any form of exercise within the past three months. Current smokers were defined as individuals who reported smoking cigarettes regularly at the time of the survey.

For blood sample processing from Taiwan Biobank participants, 10–15 milliliters of blood were collected from each individual into ethylenediaminetetraacetic acid tubes, which were kept at 4 °C until centrifugation. Samples were centrifuged at approximately 2800 rpm for 10 min to separate plasma, buffy coat, and red blood cell fractions. Each fraction was then aliquoted into cryotubes (typically 100 µL to 1 mL per tube), organized in storage boxes, and stored at −80 °C. Biochemical measurements comprised fasting plasma glucose, HbA1C, HDL-C, LDL-C, and triglycerides. Serum YKL-40 concentrations were determined using commercially available ELISA kits (R&D, Minneapolis, MN, USA). The intra- and inter-assay coefficients of variation were 6.7% and 7.7%, respectively.

### 4.3. Surrogate Markers of Insulin Resistance

Although the hyperinsulinemic-euglycemia clamp remains the gold standard for assessing insulin resistance [31], its widespread clinical application is limited due to high cost, procedural complexity, invasiveness, and ethical considerations. To overcome these limitations, the homeostatic Model Assessment of Insulin Resistance (HOMA-IR) is commonly employed. However, insulin measurement is not routinely accessible in many clinical settings. Recent studies have investigated simpler methods to predict insulin resistance using routine laboratory tests. Among these, the triglyceride-glucose (TyG) index, calculated as the product of triglyceride and fasting plasma glucose, has demonstrated high sensitivity and specificity relative to both HOMA-IR and hyperinsulinemic-euglycemic clamp test [32,33]. Additionally, obesity-related measures such as WC, waist-to-height ratio, and BMI are frequently utilized due to their ease of measurement and established associations with insulin resistance. Our previous research compared the effectiveness of TyG index, TyG-WC, and TyG-BMI in detecting insulin resistance [34]. The results indicated that TyG-BMI was the most effective surrogate marker for early detection of insulin resistance in Taiwanese individuals without diabetes. Furthermore, TyG-BMI strongly predicted new-onset T2DM in a large cohort of 116,858 Chinese individuals without diabetes, with a median follow-up period of 2.98 years. Participants in the highest quartile of TyG-BMI exhibited an 8.468-fold increased risk for developing new-onset T2DM compared to those in the lowest quartile [35]. Hence, TyG-BMI was selected as a surrogate marker of insulin resistance in the current study. Participants in the highest quartile of TyG-BMI were classified as insulin-resistant. TyG-BMI was calculated as follows: Ln [TG (mg/dL) × FPG (mg/dL)/2] × BMI (kg/m^2^).

### 4.4. Definition of Metabolic Syndrome and Obesity

Metabolic syndrome was defined according to the American Heart Association/National Heart, Lung and Blood Institute (AHA/NLHBI) criteria, using Asian-specific thresholds for abdominal obesity [6]. In accordance with the study protocol, individuals with a known history of diabetes or those taking lipid-lower medications were excluded from the analysis. Consequently, metabolic syndrome was diagnosed if participants exhibited at least three of the following conditions: (1) WC ≥ 90 cm for males and ≥ 80 cm for females; (2) SBP ≥ 130 mmHg, DBP ≥ 85 mmHg, or current use of antihypertensive medication; (3) fasting plasma glucose ≥ 5.6 mmol/L; (4) HDL-C < 1.03 mmol/L in males and <1.30 mmol/L in females; (5) triglycerides ≥ 1.7 mmol/L. Obesity was categorized based on the Asian-Pacific BMI cutoff points: normal weight (18.5–22.9 kg/m^2^), overweight (23–24.9 kg/m^2^), and obese (≥25 kg/m^2^) [36].

### 4.5. Statistical Analysis

Participants were stratified into quartiles based on their serum YKL-40 levels. Clinical characteristics were presented as means ± standard deviation or as percentages. For variables with markedly skewed distributions, medians and interquartile ranges were reported instead. Comparisons of continuous variables among quartiles were conducted using analysis of variance (ANOVA), while categorical variables were assessed via the chi-square test. Post hoc analyses following ANOVA were performed using the Bonferroni method. Pearson’s correlation coefficients were calculated to assess associations between TyG-BMI and various clinical and biochemical parameters, including YKL-40 levels. Variables demonstrating significant correlations with TyG-BMI in univariate analysis were further evaluated using stepwise multivariate linear regression to identify independent predictors of TyG-BMI. Logistic regression was performed to calculate odds ratios (ORs) and 95% confidence intervals (CIs) for the prevalence of TyG-BMI-estimated insulin resistance and metabolic syndrome across YKL-40 quartiles. Additionally, interaction effects between overweight/obesity and YKL-40 levels on metabolic syndrome prevalence were tested. All statistical analyses were conducted using IBM SPSS Statistics version 24.0 (IBM Corp., Armonk, NY, USA). Statistical significance was set at a two-sided *p* value less than 0.05.

## 5. Conclusions

Our study demonstrated a strong association between YKL-40 levels and TyG-BMI-estimated insulin resistance in a Chinese population without T2DM, independent of central obesity. Furthermore, elevated YKL-40 levels were linked to a higher prevalence of metabolic syndrome among both normal weight and overweight/obesity participants. Notably, we observed a significant interaction effect between overweight/obesity and YKL-40 levels in relation to the prevalence of metabolic syndrome. These findings enhance our understanding of the potential pathophysiological role of YKL-40 levels in cardiometabolic disorders and in modulating long-term clinical outcomes in patients with coronary artery disease.

## Figures and Tables

**Table 1:** Baseline characteristics of participants across quartiles of YKL-40 levels.

	Q1, n = 1076	Q2, n = 1076	Q3, n = 1076	Q4, n = 1076
Age (years)	42.3 ± 9.7	45.3 ± 10.0 ^b^	48.9 ± 10.3 ^b,d^	53.8 ± 10.0 ^b,d^
Male (n) (%)	444 (41.3%)	472 (43.9%)	460 (42.8%)	477 (44.3%)
Smoker (n) (%)	195 (18.1%)	188 (17.5%)	196 (18.2%)	199 (18.5%)
Exercise habits (n) (%) ^‡^	370 (34.4%)	412 (38.3%)	448 (41.7%)	523 (48.6%)
Body mass index (kg/m^2^)	23.73 ± 3.45	23.91 ± 3.54	23.97 ± 3.44	24.10 ± 3.47
Waist circumference (cm)	81.38 ± 9.58	82.10 ± 9.61	82.70 ± 9.41 ^a^	83.76 ± 9.86 ^b,d^
Systolic BP (mmHg) *	111.5 ± 13.8	112.4 ± 14.6	112.7 ± 15.5	116.8 ± 16.5 ^b,d^
Diastolic BP (mmHg) *	70.6 ± 9.8	70.9 ± 10.3	70.9 ± 10.6	72.0 ± 10.5 ^a^
Fasting glucose (mmol/L)	5.01 ± 0.38	5.07 ± 0.41	5.12 ± 0.44 ^b,c^	5.14 ± 0.43 ^b,d^
HbA1C (%)	5.45 ± 0.32	5.51 ± 0.33 ^b^	5.56 ± 0.35 ^b,c^	5.58 ± 0.35 ^b,d^
Cholesterol (mmol/L)	4.94 ± 0.87	4.99 ± 0.87	5.02 ± 0.91	5.05 ± 0.98 ^a^
Triglycerides (mmol/L)^†^	0.85 (0.61–1.23)	0.94 (0.70–1.34) ^b^	1.04 (0.76–1.48) ^b,d^	1.19 (0.85–1.72) ^b,d^
HDL-C (mmol/L)	1.46 ± 0.34	1.43 ± 0.34	1.41 ± 0.34 ^a^	1.40 ± 0.34 ^a^
LDL-C (mmol/L)	3.10 ± 0.87	3.15 ± 0.80	3.16 ± 0.80	3.10 ± 0.81
TyG-BMI	194.6 ± 35.8	199.2 ± 37.1 ^a^	201.8 ± 35.7 ^b^	206.3 ± 37.6 ^b,d^
Metabolic syndrome (n) (%) ^‡^	98 (9.1%)	134 (12.5%)	166 (15.4%)	200 (18.6%)
Component of metabolic syndrome ^§^				
Elevated waist circumference ^‡^	368 (34.2%)	403 (37.5%)	456 (42.4%)	478 (44.4%)
Elevated triglycerides ^‡^	113 (10.5%)	147 (13.7%)	205 (19.1%)	286 (26.6%)
Reduced HDL-cholesterol	206 (19.1%)	230 (21.4%)	244 (22.7%)	241 (22.4%)
Elevated blood pressure ^‡^	192 (17.8%)	225 (20.9%)	256 (23.8%)	346 (32.2%)
Elevated fasting glucose ^‡^	90 (8.4%)	144 (13.4%)	188 (17.5%)	177 (16.4%)

Abbreviations: BP, blood pressure; HDL-C, high-density lipoprotein cholesterol; HbA1C, glycated hemoglobin; LDL-C, low-density lipoprotein cholesterol; TyG-BMI, triglyceride-glucose body mass index. * Participants with antihypertensive drug treatment were excluded from the analysis. ^†^ Data with skew distribution are presented as median (interquartile range) and logarithmically transformed before statistical testing to meet the assumption of normal distribution. ^§^ Participants were considered to have metabolic syndrome if at least three of the following features were observed: (1) abdominal obesity (waist circumference ≥ 90 cm in men or ≥80 cm in women); (2) hypertriglyceridemia (triglycerides ≥ 150 mg/dL [1.7 mmol/L]); (3) low HDL-cholesterol (<40 mg/dL [1.03 mmol/L] for men, <50 mg/dL [1.30 mmol/L] for women); (4) elevated blood pressure (systolic ≥ 130 mmHg, diastolic ≥ 85 mmHg, or current use of antihypertensive medication); or (5) elevated fasting glucose (≥100 mg/dL [5.6 mmol/L]). ^‡^ *p* < 0.001 for א^2^–test. ^a^ *p* < 0.05 versus Q1 by Bonferroni method. ^b^ *p* < 0.001 versus Q1 by Bonferroni method. ^c^ *p* < 0.05 versus Q2 by Bonferroni method. ^d^ *p* < 0.001 versus Q2 by Bonferroni method.

**Table 2:** Stepwise linear regression: Univariate and multivariate analyses of predictors of TyG-BMI.

	Univariate Analysis		Multivariate Analysis			
Variables	Standardized β	*p* Value		Standardized β	*p* Value	Cumulative R2
Age	0.048	0.002	WC	0.714	0.0E0 ^‡^	0.669
Male gender	0.311	2.45 × 10^−90^	HDL-C	−0.189	3.32 × 10^−84^	0.697
Smoking	0.158	1.18 × 10^−23^	Diastolic BP ^§^	0.083	1.85 × 10^−9^	0.705
Exercise habits	0.017	0.273	Male gender	−0.076	5.08 × 10^−15^	0.709
WC	0.8178	0.0E0 ^‡^	LDL-C	0.074	7.04 × 10^−17^	0.713
Systolic BP ^§^	0.348	1.84 × 10^−114^	YKL-40 level *	0.059	1.07 × 10^−10^	0.715
Diastolic BP ^§^	0.379	4.40 × 10^−137^	Age	−0.061	8.94 × 10^−10^	0.717
HDL-C	−0.475	1.75 × 10^−224^	Systolic BP ^§^	0.042	0.003	0.717
LDL-C	0.242	2.49 × 10^−54^				
YKL-40 level *	0.108	6.88 × 10^−12^				

Abbreviations: TyG-BMI, triglyceride-glucose body mass index; WC, waist circumference; BP, blood pressure; HDL, high-density lipoprotein; LDL, low-density lipoprotein. * YKL-40 levels were log-transformed prior to analysis to meet the assumption of normality. ^§^ Participants with antihypertensive medications were excluded from the analysis to avoid the effect of medication while analyzing the association of YKL-40 level with the variables of interest. ^‡^ 0.0E0 represents *p* < 1.0 × 10^−307^, used to denote extremely significant results.

**Table 3:** Logistic regression analysis for TyG-BMI-estimated insulin resistance across YKL-40 quartiles.

	1st Quartile	2nd Quartile		3rd Quartile		4th Quartile	
		OR (95% CI)	*p* Value	OR (95% CI)	*p* Value	OR (95% CI)	*p* Value
Model 1	Reference	1.165 (0.949–1.430)	0.144	1.389 (1.136–1.698)	0.001	1.684 (1.382–2.051)	2.23 × 10^−7^
Model 2	Reference	1.171 (0.947–1.457)	0.145	1.470 (1.189–1.818)	3.78 × 10^−4^	1.839 (1.475–2.291)	5.94 × 10^−8^
Model 3	Reference	1.182 (0.897–1.557)	0.234	1.605 (1.216–2.117)	0.001	1.943 (1.453–2.598)	7.00 × 10^−6^
Model 4	Reference	1.140 (0.851–1.527)	0.379	1.589 (1.186–2.130)	0.002	2.006 (1.477–2.725)	8.00 × 10^−5^

Model 1: Unadjusted. Model 2: Adjusted for age, sex, smoking and exercise habits. Model 3: Adjusted for age, sex, smoking, exercise habits and waist circumference. Model 4: Adjusted for age, sex, smoking, exercise habits, waist circumference, systolic blood pressure, diastolic blood pressure, low-density lipoprotein cholesterol and high-density lipoprotein cholesterol.

**Table 4:** Logistic regression analysis for metabolic syndrome across YKL-40 quartiles.

	1st Quartile	2nd Quartile		3rd Quartile		4th Quartile	
		OR (95% CI)	*p* Value	OR (95% CI)	*p* Value	OR (95% CI)	*p* Value
All participants							
Model 1	Reference	1.453 (1.154–1.829)	0.001	1.859 (1.487–2.324)	5.25 × 10^−8^	2.521 (2.029–3.132)	6.78 × 10^−17^
Model 2	Reference	1.301 (1.023–1.654)	0.032	1.528 (1.204–1.939)	4.97 × 10^−4^	1.798 (1.411–2.292)	2.0 × 10^−6^
Normal weight participants (BMI < 23 kg/m^2^)							
Model 1	Reference	2.020(0.848–4.809)	0.112	3.776 (1.642–8.430)	0.001	7.525 (3.514–16.114)	2.05 × 10^−7^
Model 2	Reference	1.514 (0.626–3.657)	0.357	2.208 (0.965–5.051)	0.061	2.912 (1.297–6.541)	0.010
Overweight and obese participants (BMI ≥ 23 kg/m^2^)							
Model 1	Reference	1.402 (1.086–1.810)	0.010	1.690 (1.316–2.170)	3.90 × 10^−5^	2.134 (1.669–2.728)	1.46 × 10^−9^
Model 2	Reference	1.326 (1.017–1.729)	0.037	1.528 (1.171–1.993)	0.002	1.731 (1.320–2.272)	7.50 × 10^−5^

Model 1: Unadjusted. Model 2: Adjusted for age, sex, smoking and exercise habits.

## Data Availability

The datasets used and analyzed during the current study are available from the corresponding author Yu-Lin Ko upon reasonable request.

## Abbreviations

The following abbreviations are used in this manuscript:

T2DM	Type 2 diabetes mellitus
HDL	High-density lipoprotein
WC	Waist circumference
SBP	Systolic blood pressure
DBP	Diastolic blood pressure
HbA1C	Glycated hemoglobin
BMI	Body mass index
TyG	Triglyceride-glucose
TyG-BMI	Triglyceride-glucose body mass index
LDL	Low-density lipoprotein
OR	Odds ratio
CI	Confidence interval
HOMA-IR	Homeostatic Model Assessment of Insulin Resistance
TWB	Taiwan Biobank
ELISA	Enzyme-linked immunosorbent assay (ELISA) kits
ANOVA	Analysis of variance
